# Risk Factors for HSV-2 Infection among Sexual Partners of HSV-2/HIV-1 Co-Infected Persons

**DOI:** 10.1186/1756-0500-4-64

**Published:** 2011-03-15

**Authors:** Andrew Mujugira, Amalia S Magaret, Jared M Baeten, Connie Celum, Jairam Lingappa

**Affiliations:** 1 Department of Global Health, University of Washington, Seattle, WA, USA; 2 Department of Laboratory Medicine, University of Washington, Seattle, WA, USA; 3 Department of Medicine, University of Washington, Seattle, WA, USA; 4 Department of Epidemiology, University of Washington, Seattle, WA, USA; 5 Department of Pediatrics, University of Washington, Seattle, WA, USA

## Abstract

**Background:**

Herpes simplex virus type 2 (HSV-2) is the most frequent cause of genital ulcer disease worldwide and has been associated with increased risk for HIV-1 acquisition and transmission. We conducted a cross-sectional analysis of risk factors for HSV-2 infection among HIV-1 uninfected partners, whose partners were co-infected with HIV-1 and HSV-2.

**Methods:**

Between November 2004 and April 2007, 3408 HIV-discordant couples, in which the HIV-1 infected partners were HSV-2 seropositive with CD4 250 cells/mm^3 ^or greater, were enrolled in an HSV-2 suppression trial to prevent HIV-1 transmission at 14 sites in 7 African countries. Clinical & behavioral data, HSV-2 and HIV-1 testing were conducted at enrolment. Univariate and multivariate Poisson regression analyses were performed separately, by gender of the HIV-1 infected partner.

**Results:**

Among 3354 HIV-1 uninfected participants, 32% were female and overall 71% were HSV-2 seropositive. Among couples with female HIV-1 infected partners, HIV-1 plasma RNA [aPR 1.03; 95% CI: 0.99 to 1.06; p = 0.11] and CD4 count [aPR 1.00; 95% CI: 0.98 to 1.01; p = 0.48] in the HSV-2/HIV-1 dually infected female and circumcision in the HIV-1 uninfected male partner [aPR 0.94; 95% CI: 0.88 to 1.00; p = 0.06] were not associated with reduced risk of HSV-2 seropositivity, after adjusting for other factors.

**Conclusions:**

In this cross-sectional analysis of African HIV-1 serodiscordant heterosexual couples with prevalent HSV-2 infection in the HIV-1 infected partner, HIV-1 plasma RNA and CD4 count in the dually-infected partner and male circumcision in the HIV-1 uninfected partner were not associated with HSV-2 concordance.

**Trial Registration:**

ClinicalTrials.gov NCT00194519

## Background

Herpes simplex virus type 2 (HSV-2) is the most common cause of genital ulcer disease (GUD) worldwide. HSV-2 prevalence in sub-Saharan Africa ranges from 27-57% in men to 30-74% in women [1,2] and is higher in HIV-1 infected persons [3]. Epidemiologic studies suggest synergy between HIV-1 and HSV-2 that facilitates the spread of both viruses, with HSV-2 increasing HIV-1 susceptibility and infectiousness [4] and HIV-1 infection increasing HSV-2 reactivation frequency [5].

The increased risk of HIV-1 transmission and acquisition due to HSV-2 infection in the context of an overall high risk of HIV-1 transmission among stable, heterosexual HIV-1 serodiscordant couples (one partner HIV-1 infected and the other HIV-1 uninfected) [6], which are highly prevalent in Africa [7], make evaluation of risk factors for HSV-2 within HIV-1 serodiscordant couples from diverse parts of Africa of interest.

We conducted a cross-sectional analysis of risk factors for HSV-2 seropositivity in >3300 HIV-1 uninfected members of African HIV-1 serodiscordant couples in which the HIV-1 infected partner was dually-infected with HSV-2. In particular, we sought to assess whether HIV-1 plasma RNA level or CD4 count in the HIV-1 infected partner, as indicators of higher HIV-1 infectiousness and more advanced immunosuppression, modifies HSV-2 infectiousness, and if male circumcision in the HIV-uninfected partners of women with HSV-2/HIV-1 dual infection protected them from HSV-2 acquisition.

## Methods

### Study Population

From November 2004 to April 2007, 3408 heterosexual HIV-1 discordant couples from 14 sites in Botswana, Kenya, Rwanda, South Africa, Tanzania, Uganda and Zambia were enrolled into the Partners in Prevention HSV/HIV Transmission Study, a randomized clinical trial of suppressive acyclovir therapy to reduce sexual HIV-1 transmission from HSV-2/HIV-1 dually-infected women and men to their HIV-1 uninfected partners [7]. HIV-1 discordant couples were eligible for enrolment into the trial if they were sexually active and planning to remain partnered for at least 24 months and if the HIV-1-infected participant was HSV-2 infected, had a CD4 count 250 cells/mm^3 ^or greater, was not on antiretroviral therapy, and was not pregnant. HIV-1 uninfected members of the discordant couples could be either HSV-2 seropositive or seronegative. Clinical and behavioral data collected at study enrolment were used for this analysis.

### Laboratory Methods

HIV-1 testing was done using 2 rapid assays in parallel, most commonly Determine HIV-1/2 (Abbott Diagnostics), Uni-Gold HIV-1 (Trinity Biotech), or Capillus HIV-1/HIV-2 (Trinity Biotech) and paired positive rapid assay results were confirmed by HIV-1 EIA [7]. At all study sites, HSV-2 serostatus was determined using HerpeSelect HSV-2 gG2-based EIA (Focus Technologies). Given the results of previous studies using this assay in African cohorts, a cut-off value of ≥3.5 was chosen to define seropositivity, in order to improve test specificity [8-10]. CD4 quantification was performed for HIV-1 infected participants at 6-month intervals using standard flow cytometry. Plasma HIV-1 RNA was quantified using the COBAS Ampliprep/COBAS TaqMan real-time HIV-1 RNA assay, version 1.0 (Roche Diagnostics, Indianapolis, IN). All HIV-1 and HSV-2 serologic results were confirmed by Western blot, performed at the University of Washington [11].

### Statistical Analysis

Data were entered onto standard case report forms and faxed to a central database at the University of Washington. Analysis was done using SAS version 9.1 (SAS Institute, Cary, NC). Univariate and multivariate analyses were performed using Poisson regression with HSV-2 status of the partner participant as the outcome variable. In order to examine male circumcision and to allow for other possible gender differences in transmission risk, separate analyses were performed for male and female HIV-1 infected participants. Backward elimination, starting with all variables significant at p < 0.1 in univariate regression, was performed to select a final multivariate model.

### Ethical Approval

Institutional review boards at the University of Washington and at all collaborating site organizations approved study procedures. All participants provided written informed consent. The trial was registered through ClinicalTrials.gov (NCT00194519).

## Results

### Characteristics of study population

Of 3408 enrolled HIV-1-uninfected participants, 3354 were eligible for this analysis (54 were excluded because either they or their partner were ineligible for the study, based on confirmatory HIV-1 or HSV-2 testing). HIV-1 seronegative partners had a median age of 35 years and 1084 (32%) were female. Couples reported a median of 4 sex acts in the month prior to enrollment (IQR 2-8). The seroprevalence of HSV-2 among HIV-1 seronegative partners was 71% overall; 63% in men versus 89% in women (p < 0.001). By study design, all HIV-1 infected partners were co-infected with HSV-2. Median CD4 count among HIV-1 infected partners was 462 cells/mm^3 ^(IQR 347-631) and median plasma HIV-1 RNA was 4.1 log_10 _copies/mL (IQR 3.3-4.7).

### Risk factors for HSV-2 infection among HIV-1 uninfected partners

Among male HIV-1 uninfected partners of HIV-1/HSV-2 dually infected females, older age (adjusted prevalence ratio [aPR] 1.11, p < 0.001), a greater number of children (aPR 1.02 per 1 child increase, p < 0.001) and greater number of years living together (aPR 1.07, per 1 year increase, p = 0.009) were significantly associated with HSV-2 infection (Table 1). Characteristics of the female HIV-1 infected partner associated with HSV-2 infection in their male HIV-1 uninfected partners included having herpetic lesions observed on genital exam at enrollment (aPR 0.92, p = 0.04), other sexual partners (aPR 1.28, p = 0.05), and using vaginal drying agents (aPR 1.08, p = 0.05). Circumcised HIV-1 uninfected men were at slightly decreased risk for HSV-2 (aPR 0.94, p = 0.06), although this did not achieve statistical significance.

**Table 1:** Population characteristics and risk factors for HSV-2 seropositivity among HIV-1 uninfected members of African HIV-1 serodiscordant partnerships

	HIV-1 uninfected female partners of males dually-infected with HSV-2 and HIV-1 (N = 1084)						HIV-1 uninfected male partners of females dually-infected with HSV-2 and HIV-1 (N = 2270)					
	
	HSV-2 negative (n = 123) # (%) or median (IQR)	HSV-2 positive (n = 961) # (%) or median (IQR)	Prevalence ratio (95% CI)	p-value	Adjusted Prevalence Ratio^#^ (95% CI)	p-value	HSV-2 negative (n = 841) # (%) or median (IQR)	HSV-2 positive (n = 1429) # (%) or median (IQR)	Prevalence ratio (95% CI)	p-value	Adjusted Prevalence ratio^#^ (95% CI)	p-value
**Characteristics of HIV-1/HSV-2** **dually-infected partner**												

Genital lesions at enrollment	27 (22%)	233 (24.2%)	1.01 (0.97, 1.07)	0.6			202 (24%)	287 (20.1%)	0.92 (0.85, 0.99)	0.028	0.92 (0.85, 0.99)	0.04

CD4 count	424 (335-566)	424 (333-571)	1.00 (0.99, 1.01)*	0.66			484 (358-669)	482 (354-659)	1.00 (0.98, 1.01)*	0.48		

Plasma HIV-1 RNA	4.35 (3.71-4.95)	4.32 (3.67-4.87)	0.99 (0.97, 1.02)	0.64			3.92 (3.13-4.50)	3.96 (3.25-4.55)	1.03 (0.99, 1.06)	0.11		

Has any other sexual partners	9 (7.3%)	78 (8.1%)	1.01 (0.94, 1.09)	0.76			7 (0.8%)	26 (1.8%)	1.26 (0.99, 1.59)	0.058	1.28 (1.00, 1.63)	0.047

**Characteristics of HIV-1 uninfected** **partner**												

Age	26 (22-30)	31 (26-39)	1.09 (1.06, 1.12)^† ^	<.001	1.08 (1.06, 1.11)^† ^	<.001	32 (28-39)	37 (31-44)	1.17 (1.14, 1.21)^† ^	<.001	1.11 (1.06, 1.15)	<.001

Years of education	8 (7-12)	8 (6-10)	0.88 (0.83, 0.93)^† ^	<.0001	0.90 (0.85, 0.96)^† ^	0.0011	9 (7-12)	9 (7-12)	0.89 (0.82, 0.97)^† ^	0.01	!	

Has any other sexual partners	1 (1.5%)	4 (0.8%)	0.91 (0.65, 1.28)	0.59			31 (7.9%)	67 (10.0%)	1.09 (0.94, 1.27)	0.27		

**Gender-specific characteristics**												

Male partner is circumcised (physical exam)	37 (30.1%)	329 (34.3%)	1.02 (0.98, 1.07)	0.36			483 (57.4%)	760 (53.2%)	0.94 (0.88, 1.00)	0.049	0.94 (0.88, 1.00)	0.0614

Female partner uses hormonal contraceptives	18 (14.6%)	157 (16.3%)	1.01 (0.96, 1.07)	0.63			160 (19.0%)	279 (19.5%)	1.01 (0.93, 1.10)	0.77		

Female uses vaginal drying	24 (19.5%)	196 (20.4%)	1.01 (0.95, 1.06)	0.82			156 (18.5%)	322 (22.5%)	1.09 (1.01, 1.18)	0.025	1.08 (1.00, 1.17)	0.051

**Couple characteristics**^‡^												

Couple is married	94 (76.4%)	776 (80.7%)	1.03 (0.98, 1.09)	0.26			589 (70.0%)	1078 (75.4%)	1.11 (1.03, 1.20)	0.005		

Duration of sexual relationship	4 (2-8)	7 (3-15)	1.06 (1.04, 1.09)^† ^	<.001			4 (2-7)	6 (3-10)	1.18 (1.13, 1.24)†	<.001		

Years living together	3 (1-6)	6 (3-13)	1.06 (1.03, 1.09)^† ^	<.001			3 (1-6)	5 (2-10)	1.20 (1.15, 1.26)†	<.001	1.07 (1.02, 1.13)	0.009

Number of children	1 (1-3)	3 (1-4)	1.03 (1.02, 1.04)	<.001			2 (1-3)	2 (1-4)	1.05 (1.04, 1.07)	<.001	1.02 (1.01, 1.04)	0.001

Informal housing	50 (41.3%)	348 (37.5%)	0.98 (0.94, 1.03)	0.41			346 (42.5%)	540 (38.9%)	0.95 (0.89, 1.01)	0.099		

Number of sex acts with study part-ner in previous month (median, IQR)	3 (2-7)	4 (2-8)	1.02 (0.99, 1.05)†	0.26			4 (2-8)	4 (2-8)	0.96 (0.92, 1.01)^† ^	0.13		

Any sex acts with partner in previous month	114 (92.7%)	885 (92.1%)	0.99 (0.92, 1.07)	0.82			796 (94.6%)	1342 (93.9%)	0.95 (0.83, 1.09)	0.47		

Any unprotected sex with study partner in previous month	32 (26.0%)	259 (27.0%)	1.01 (0.96, 1.05)	0.83			245 (29.1%)	451 (31.6%)	1.04 (0.97, 1.12)	0.23		

Enrollment site in Eastern (vs. southern) Africa	78 (63.4%)	593 (61.7%)	0.99 (0.95, 1.04)	0.71			509 (60.5%)	874 (61.2%)	1.01 (0.95, 1.08)	0.76		

**^# ^**Multivariate risk ratios, adjusted for other factors in the model as shown.

* Risk ratio of HSV2 positivity for every 100 ct increase in CD4.

† Risk ratio of HSV2 positivity for every increase of 10.

‡ As reported by HIV-1 uninfected participant.

(Note: Length of sexual relationship strongly associated with age of partner, rho = 0.66, p < .0001.)

**! **Years of education was not a significant risk factor for HSV-2 seropositivity in male HIV-1 uninfected partners

Among female HIV-1 uninfected partners of HIV-1/HSV-2 dually-infected males, only older age (aPR 1.08, p < 0.0001) and greater number of years of education (aPR 0.90, p = 0.001) of the HIV-1 infected partner were associated with HSV-2 seropositivity.

Notably, neither HIV-1 plasma RNA level [aPR 1.03; 95% CI: 0.99 to 1.06; p = 0.11] nor CD4 count [aPR 1.00; 95% CI: 0.98 to 1.01; p = 0.48], factors that might indicate increased HSV-2 infectiousness [12], were associated with increased risk of HSV-2 infection in HIV-1 uninfected partners.

## Discussion

In this multi-national study of 3354 African HIV-1 serodiscordant couples in which one partner was dually-infected with HIV-1 and HSV-2, HIV-1 plasma RNA and CD4 count of the HIV-1 infected partner were not significantly associated with HSV-2 infection concordance in the HIV-1 uninfected partner. Furthermore, despite a recent prospective clinical trial reporting association of male circumcision with reduced HSV-2 acquisition [13], male circumcision in male HIV-1 uninfected partner was only marginally protective against HSV-2 infection in our cross-sectional multivariate analysis.

Prevalence of HSV-2 in the HIV-1 uninfected partner was high in our study (63% in men and 89% in women) compared to other African cohorts [14,15], possibly due to the high rate of HSV-2 transmission reported in stable, long-term partnerships [16,17]. Also consistent with other studies [18-20], HSV-2 infection at enrollment in the HIV-1 uninfected partner was significantly associated with older age and was higher among women compared to men. The former may indicate that age is a surrogate for duration of sexual exposure in these couples [21].

A limitation of this cross-sectional study was the inability to determine temporal relationships of HIV-1 and HSV-2 infection in the dually-infected partner or recency of HSV-2 infection in the HIV-uninfected partners, thus limiting our analysis of behavioral correlates with HSV-2 infection. HSV-2 concordance may reflect HSV-2 transmission within the couple or acquisition outside the partnership. Our study eligibility criteria included that couples be sexually active and intend to remain together; as such we may have selected for older, more stable couples.

## Conclusions

In summary, among African HIV-1 serodiscordant couples in which all the HIV-infected partners were dually infected with HSV-2, 71% of their HIV-1 uninfected partners had HSV-2 infection, and HSV-2 infection in the HIV-uninfected partner was not associated with HIV-1 plasma RNA and CD4 count in the HIV-infected partner, or male circumcision in the HIV-1 uninfected partner. Given the need to identify interventions to reduce HSV-2 transmission, future research should focus on prospective analysis of risk factors for HSV-2 transmission, which will be conducted in this cohort.

## Competing interests

The authors declare that they have no competing interests.

## Authors' contributions

AM wrote the first draft along with JL and JMB. ASM analyzed the data. All authors contributed to the gathering of data, interpretation of results, and writing of the manuscript, and all approved the final draft.
